# The effects of combined dynamic navigation system and dental operating microscope on the accuracy and efficiency of fiber post removal: an in vitro study

**DOI:** 10.1186/s12903-025-05887-6

**Published:** 2025-04-03

**Authors:** Sirinya Kulvitit, Thamasorn Eamtanaporn, Phonkit Sinpitaksakul, Patcharapit Promoppatum, Suppakrit Khrueaduangkham, Thantrira Porntaveetus

**Affiliations:** 1 Department of Operative Dentistry, Faculty of Dentistry, Chulalongkorn University, Bangkok, 10330 Thailand; 2 Center of Excellence in Genomics and Precision Dentistry, Department of Physiology, Faculty of Dentistry, Chulalongkorn university, Bangkok, 10330 Thailand; 3 Department of Radiology, Faculty of Dentistry, Chulalongkorn University, Bangkok, 10330 Thailand; 4 Department of Mechanical Engineering, Faculty of Engineering, King Mongkut’s University of Technology Thonburi (KMUTT), Bangkok, 10140 Thailand

**Keywords:** Guided endodontics, Real-time navigation, Dynamic computer-assisted navigation, Dental Microscope, Fiber post removal, Ultrasonic, Root canal retreatment

## Abstract

**Background:**

There is limited information concerning the effectiveness of the combined dynamic navigation system (DNS) and dental operating microscope (DOM) technique in fiber post removal. The aim of this study was to assess the effectiveness of the DNS-DOM technique for fiber post removal compared with the freehand technique (FH)-DOM.

**Methods:**

In a phantom head-based study, 30 human mandibular teeth were utilized, with 7 mm of fiber post left in obturated canals. Under a DOM, an experienced endodontist, employing an ET18D ultrasonic tip under a rubber dam, removed the fiber posts using either FH or DNS. Pre- and post-operative CBCT scans were taken, and 3D models were reconstructed with Materialise Mimics software. Accuracy (deviation volume, distance, angle, position) and efficiency (procedure time) parameters were analyzed. Normality was assessed with the Shapiro-Wilk test, utilizing the independent samples t-test for normally distributed data and the Mann-Whitney U test for non-normally distributed data.

**Results:**

The DNS-DOM and FH-DOM groups demonstrated comparable results in deviation volume, percent deviation volume, maximum deviation distance, deviation angle, and maximum deviation position—all accuracy-related factors. We noted higher deviation and percent deviation volumes in DNS-DOM, which were not significant, compared with FH-DOM in incisors. Conversely, in molars, FH-DOM exhibited higher values than DNS-DOM, suggesting that the free-hand technique may result in less deviation under better visibility. There was a consistent trend of a higher deviation angle for DNS-DOM compared with FH-DOM across all tooth types. DNS-DOM and FH-DOM displayed a higher deviation angle in molars than in premolars and incisors. The distance between maximum deviation points and initial drilling points was smaller in molars compared with incisors and premolars. Regarding efficiency, the DNS-DOM group demonstrated a significantly longer procedure time (8 min) compared with FH-DOM. Fiber post removal time followed a similar trend in both groups, being fastest in molars, followed by premolars and incisors.

**Conclusions:**

DNS-DOM showed accuracy comparable to FH-DOM in fiber post removal when performed by an experienced endodontist with appropriate ultrasonic tips. However, DNS-DOM had a longer procedure time, potentially reducing efficiency due to the additional navigation system integration, demanding increased operator operating time.

## Introduction

The appropriate treatment for failed root canal treatment with a suspected intraradicular infection is determined by the root canals accessibility. When coronal access is possible, an orthograde root canal retreatment approach is selected [1]. To gain access to the root canal, the coronal restoration, post and root canal filling material must be removed. Fiber post removal is one of the most challenging procedures during radicular access, because distinguishing the fiber post from the dentin structure is difficult due to their similarity in color and hardness. One major complication after fiber post removal is severe deviation from the root axis or perforation [2–4].

The Ultrasonic technique is a frequently used option that allows dentists to remove fiber posts safely and effectively [2, 5]. Using an ultrasonic tip can facilitate fiber post removal under a dental operating microscope (DOM) due to the ultrasonic shank and tip’s smaller profile compared with the contra-angle handpiece, improving visibility under the DOM [6].

The dynamic navigation system (DNS) is also used to optimize the accuracy of many dental procedures, including endodontic treatment, such as locating calcified canals, fiber post removal, and microsurgery [7–11]. The Implant Real-time Imaging System (IRIS-100, EPED Inc., Taiwan) is a type of DNS that has superior accuracy compared with the Freehand (FH) technique in dental implant placement [12]. In addition to implant placement, IRIS-100 DNS can also be used for fiber post removal because the software can designate the fiber post’s location, axis, and size similar to the way an implant is planned. Moreover, IRIS-100 DNS can be used clinically when an ultrasonic instrument and rubber dam are required. However, there has been no clinical investigation performed with IRIS-100 DNS when ultrasonic and rubber dam techniques are utilized.

A previous study using X-guide software (X-Nav Technologies, Lansdale, PA, USA) in fiber post removal found that this approach can offer more efficient fiber post removal compared with the FH technique with DOM in the absence of a rubber dam. The study determined the accuracy of the DNS by removing fiber posts according to the preplanned trajectory and complete guidance of the system [6]. This approach is advantageous for assessing the accuracy of the DNS alone. However, in real-life situations, recent technology like the DNS is often employed in conjunction with a DOM rather than a standalone instrument replacing the DOM. This method enables the operator to double check the accuracy of DNS with DOM to ensure accuracy in fiber post removal.

There is currently no report concerning the accuracy and efficiency of the combined DNS-DOM technique compared with the FH technique, using only DOM for fiber removal. Thus, the aims of this study were (1) to compare the accuracy of the IRIS-100 DNS-DOM versus the FH-DOM technique in fiber post removal using an ultrasonic instrument and a rubber dam, and (2) to determine the efficiency of fiber post removal using the IRIS-100 DNS-DOM versus the FH-DOM technique. The null hypothesis proposed that using the DNS-DOM and the FH-DOM technique for fiber post removal demonstrates similar accuracy and efficiency.

## Materials and methods

### Study design, ethics, and sample size calculation

This study was approved by the Human Research Ethics Committee, Faculty of Dentistry, Chulalongkorn University. Because teeth were collected from the Department of Oral Surgery without personal identifiable information, informed consent was provided by the head of the department. The sample size calculation was conducted using G*Power Software (version 3.1, Germany) using the independent samples t test with a type I error of 0.05 and 0.8 power based on a previous study [13]. The final estimation for the sample size was 14 teeth for each group, which was adjusted to 15 teeth to equalize the number of teeth in 3 different categories (incisors, premolars, and molars).

**Fig. 1:**
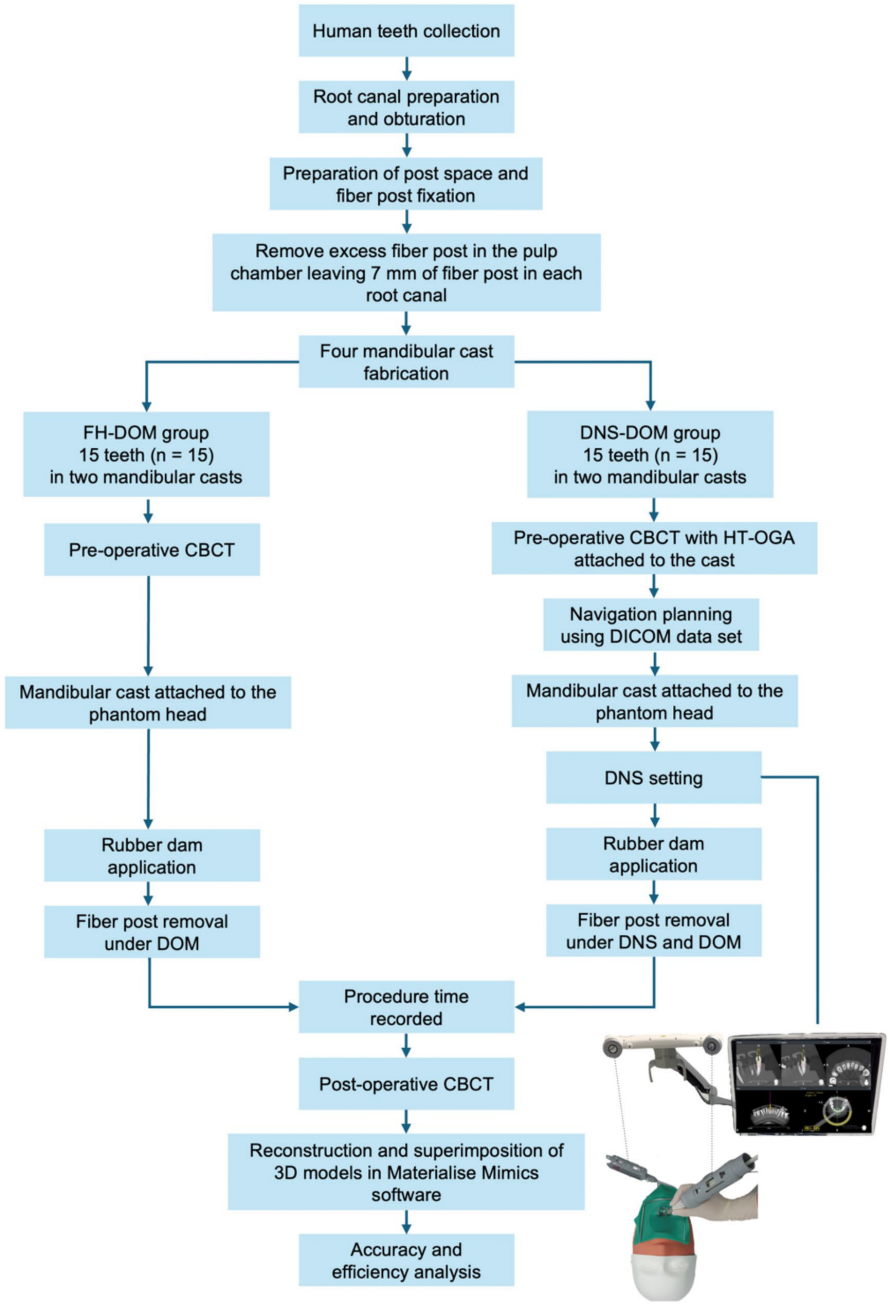
Experiment flowchart. Close-up inter-experimental photographs of the tooth were captured from the DOM (Zeiss Extaro 300) at a magnification of 2.5 (21.25-fold magnification). Other intra-experiment photographs were captured by a digital camera

Thirty extracted human mandibular teeth were collected from the Department of Oral Surgery, Chulalongkorn University without personally identifiable information. The teeth were extracted due to periodontal disease or for orthodontic purposes. The teeth were sterilized by immersing them in 10% formalin for 5 days. The soft and hard tissue remnants on the external root surfaces were removed. Bucco-lingual and mesio-distal periapical radiographs were taken. The teeth were selected according to the following inclusion and exclusion criteria. The inclusion criteria were (a) mandibular incisors and premolars with a round single straight root canal, (b) mandibular molars with a round straight distal root canal and approximately 7 mm between the distal root canal orifice and the reference point (DB cusp), (c) teeth with a canal width at 7 mm from the occlusal reference point smaller than the “D.T. Light-post finishing drill” size #1 width at 7 mm from the tip of the drill (1.2 mm), and (d) teeth with a canal width ratio less than 2 at 14 mm from the occlusal reference point in the BL/MD direction. The exclusion criteria comprised teeth with extensive caries, restorations, including filling materials and crowns; cracks, incomplete root formation, and tooth length under 18 mm and over 23 mm, and root canals with an initial apical file size larger than 35. The experimental flowchart summarizing the steps in the experiment is seen in Fig. 1. The photographs of the tooth with a fiber post and ultrasonic tip were captured from the DOM (Zeiss Extaro 300) at a magnification of 2.5 (21.25-fold magnification).

### Root canal preparation and obturation

The working length was set at 0.5 mm short of the apical foramen. The root canals were prepared using rotary files up to a size 30/0.07 (ProTaper Next^®^, Dentsply Maillefer) followed by irrigation with 10 ml 2.5% NaOCl, 10 ml 17% EDTA for 1 min, 10 ml 2.5% NaOCl, and 10 ml normal saline solution. The root canals were blotted dry with paper points and obturated with ProTaper Next^®^ X3 gutta-percha master cones (DENTSPLY) and AH Plus sealer (DENTSPLY). Gutta percha was down-packed using a continuous wave of condensation technique to 14 mm from the occlusal reference point.

### Post space preparation and fiber post fixation

A “D.T. Light-post finishing drill” size #1 was used for post space preparation to 14 mm from the occlusal reference point. The post space was irrigated with 10 ml deionized water and blotted dry using paper points. A D.T light-post Illusion X-RO size #1 (RTD, St Egreve, France) was fixed with OptiBond Solo Plus (Kerr, Orange, CA) and dual-cure NX3 universal resin cement (White) (Kerr) according to the manufacturer’s instructions. The excess fiber post in the pulp chamber was removed using a high speed tapered round-end diamond bur to leave 7 mm of fiber post in the root canal.

### Mandibular cast fabrication and experimental groups

Four mandibular casts were fabricated. Teeth were numbered 1 − 10 for each tooth type and the “Random sample” command in Excel (Microsoft Office Excel 2010; Microsoft Corporation, Redmond, WA, USA) was used to randomly assign the teeth into each group. Each group contained 15 teeth (5 incisors, 5 premolars, and 5 molars). The teeth were mounted in the mandibular cast that was placed in a phantom head during the operation. Two casts composed of fifteen samples teeth were used each group: (1) DNS-DOM group: Fiber post was removed under DOM (Zeiss Extaro 300) and real-time navigation in the guidance mode of the IRIS-100 software (beta8_IRIS_v6.4.0.0), (2) FH-DOM group: Fiber post was removed freehand under DOM. To simulate a full mandibular arch in the cast inside the phantom head, human teeth were added until each cast contained 14 teeth.

### Pre-operative Cone-Beam Computed Tomography (CBCT) scanning and patient position simulation

**Fig. 2:**
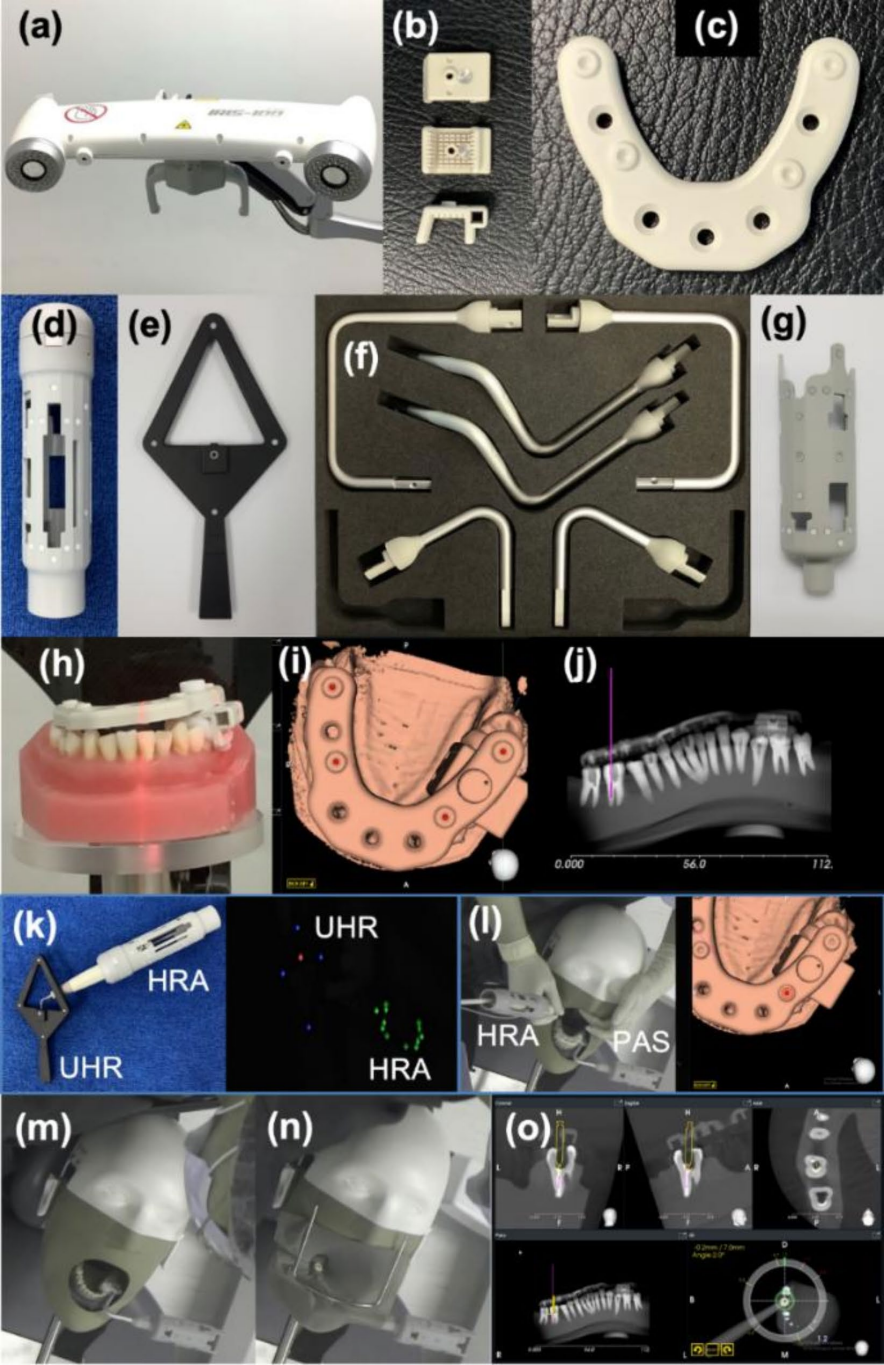
The instruments used in DNS system and digital workflow. Pre-experiment photographs in Fig. 2a − 2h captured by a digital camera. Intra-experiment photographs in Fig. 2i − 2o captured from IRIS-100 software. The instruments used in the DNS system were (**a**) Tracking unit (TU), (**b**) Holding tray (HT), (**c**) Occlusal guide appliance with four radiopaque fiducial markers (OGA), (**d**) Handpiece reflective attachment (HRA), (**e**) Ultrasonic handpiece registrator (UHR), (**f**) Jaw attachment (JA), and (**g**) Positioner arm set (PAS). (**h**) CBCT radiographic examination in mandibular casts-HT-OGA, (**i**) Feature point registration planned, (**j**) Added fiber post in the DNS plan, (**k**) Instrument registration, (**l**) Feature point registration, (**m**) HT-JA-PAS was attached to the cast, (**n**) Rubber dam was placed on the cast, and (**o**) Fiber post removal using guidance mode in the DNS system

Each mandibular cast received a pre-operative CBCT radiographic examination with a 3D Accuitomo 170 machine (J.Morita Inc, Kyoto, Japan). The CBCT radiographic examination settings were: field of view (FOV) 80 × 80 mm (voxel size 0.16 mm), tube current 5 mA, and tube voltage 80 kVp. In the DNS-DOM group, the holding tray-occlusal guide appliance with four radiopaque fiducial markers (HT-OGA) was attached on the teeth using thermoplasticized resin on the contralateral side of the same arch of the procedure during CBCT (Fig. 2h). One Volume Viewer software (J. MORITA MFG. CORP., Kyoto, Japan) was used for evaluating the fiber post’s position and angulation. Digital Imaging for Communication in Medicine (DICOM) data sets of the DNS-DOM samples were used for navigation planning prior to fiber post removal. The patient’s position in the dental chair was simulated by attaching the NISSIM phantom head (NISSIN, Kyoto, Japan) with the mandibular cast to the dental chair using a chair mount unit2 (NISSIN, Kyoto, Japan).

### IRIS-100 software DNS planning and set-up

Figure 2 displays the instruments used in the DNS system and the step-by-step approach for fiber post removal under DOM and real-time navigation. The instruments used in the DNS system (Fig. 2a-g) comprised the tracking unit (TU), holding tray (HT), occlusal guide appliance with four radiopaque fiducial markers (OGA), handpiece reflective attachment (HRA), ultrasonic handpiece registrator (UHR), jaw attachment (JA), and positioner arm set (PAS).

DICOM data sets obtained from CBCT scans of the samples were uploaded to the IRIS-100 navigation program to generate CBCT image of the tooth samples that will be planned for fiber post removal. These images were used to determine the position and dimensions of the fiber post and to facilitate the integration of fiducial markers for accurate localization. A radiopacity threshold of 300 was used in the feature point registration planning. Four feature points were assigned at the center of 4 fiducial markers on the OGA radiographic image in the software (Fig. 2i). Fiber post dimension and position were assigned according to the CBCT image (Fig. 2j). Instrument registration was done by inserting the ET18D tip (Acteon, Merignac, France) into the UHR (Fig. 2k) to link the TU with the ET18D position through HRA. Feature Point Registration (FPR) or patient registration was done by attaching the HT, OGA, JA, and PAS to the cast and touching the tip of the ET18D on 4 fiducial markers on the OGA as in the FPR plan. FPR was done to link the TU with the cast position through PAS (Fig. 2l). The OGA was removed, leaving the HT-JA-PAS attached to the cast (Fig. 2m). A rubber dam was placed (Fig. 2n).

### Fiber post removal and post-operative CBCT

Fiber post removal was done by a single operator, a six-year experienced board-certified endodontist (Si.K.). The operator was trained by removing ten fiber posts under DOM and real-time navigation in the guidance mode of the IRIS-100 software. The DOM (Zeiss Extaro 300) and the phantom head were adjusted until the fiber post was visible under DOM at a magnification of 1 (8.5-fold magnification) and an ergonomic operative area for fiber post removal was accomplished. Fiber post removal using ET18D with normal saline coolant at power 10 of P5 newtron XS ultrasonic generator. In the DNS-DOM group, fiber post removal was done under DOM and IRIS-100 DNS (Fig. 2o). The fiber post removal time was recorded by a digital stopwatch from the moment the ET18D started removing the fiber post at 7 mm from the occlusal reference point until complete fiber post removal at 14 mm from the occlusal reference point and apical gutta percha was seen. During the operation, when closer inspection was needed, DOM was adjusted to a magnification of 2.5 (21.25-fold magnification) to help distinguish the fiber post from the surrounding root canal wall. Each cast underwent a post-operative CBCT radiographic examination using the same settings as the pre-operative CBCT.

### Reconstruction and superimposition

The reconstruction and superimposition of the CBCT images were collaboratively done by two engineers (P.P. and S.K.) and a dentist (T.E.) who were blinded to the sample groups.

The analysis process began with importing the CBCT images into Mimics software (Materialise Mimics Version 25.0.1.583, Leuven, Belgium), where thresholds were selected to distinguish various dental structures. This careful segmentation allowed us to isolate the dentin, enamel, fiber post, and air space within the samples.

Following segmentation, we use the program’s tools to generate 3D models of tooth structures in both their pre- and post-treatment states. To assess the accuracy of fiber post removal, we superimposed the pre- and post-treatment 3D reconstructions using Mimics’ registration tools. This superimposition technique enabled precise comparison between the two states, allowing for detailed deviation analysis of the structures.

The final step involved quantifying the volume of dentin removed during the procedure. Using the measurement tools within Mimics, we calculated these volumetric changes. To ensure objectivity in our analysis, the measurements were independently verified through collaborative assessment by both engineers and a dentist, all of whom were blinded to the sample groupings.

### Accuracy and efficiency measurement

**Fig. 3:**
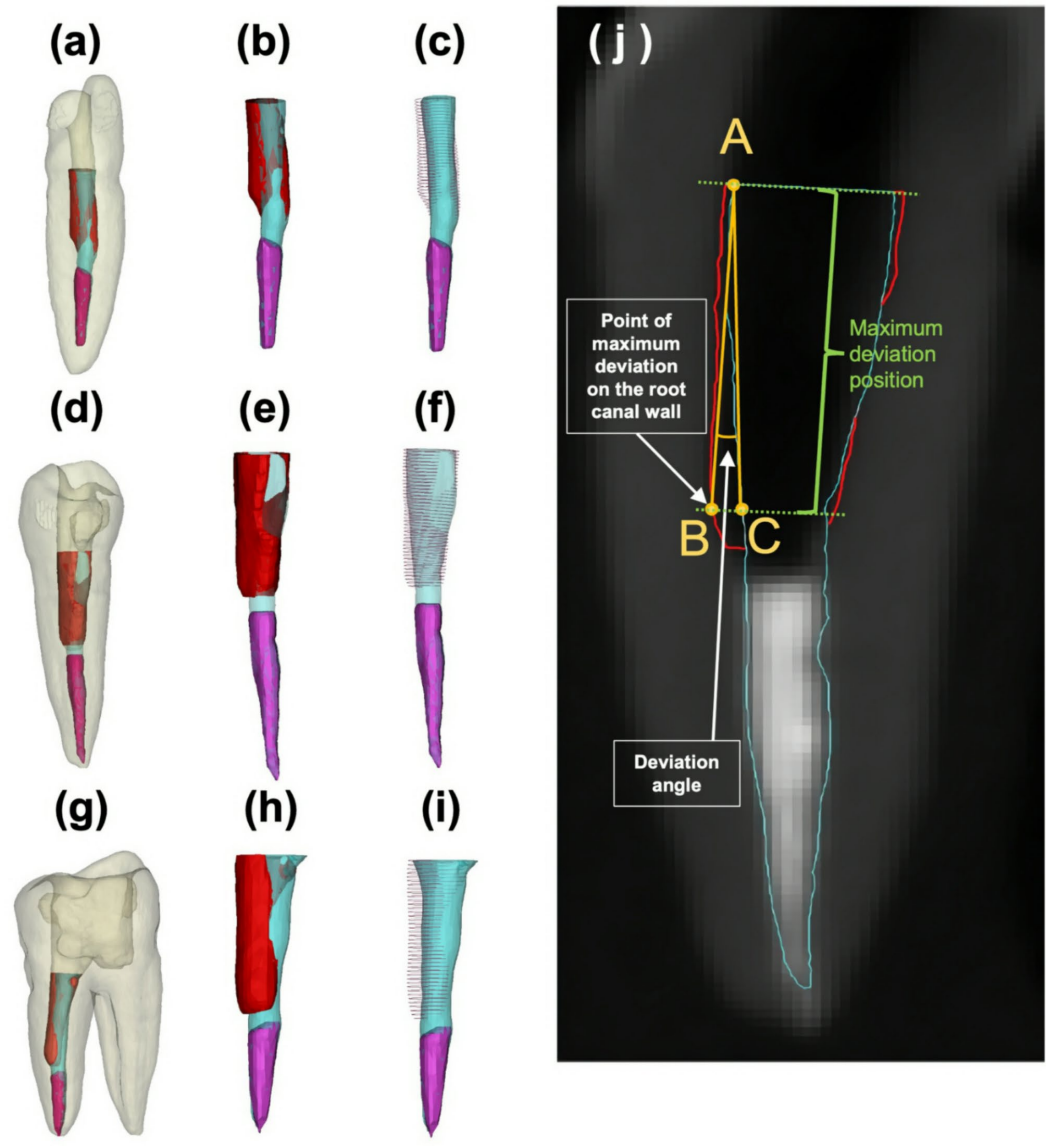
Superimposition of the reconstructed of 3-dimensional models and image of the pre-CBCT and post-CBCT images of representative samples. (**a-c**) Superimposed models of an incisor. (**d-f**) Superimposed models of a premolar. (**g-i**) Superimposed models of a molar. (**a, d, g**) Superimposed models of tooth structure (Light yellow), fiber post (Light blue), root canal filling materials (Pink), and deviation volume (Red). (**b, e, h**) Close-up superimposed models of fiber post (Light blue), root canal filling (Pink), and deviation volume (Red) without tooth structure. (**c, f, i**) Close-up superimposed models of fiber post (Light blue), root canal filling (Pink), and translucent deviation volume (Red polylines) without tooth structure. (**j**) Measurement of the maximum deviation distance, deviation angle, and maximum deviation position was illustrated. The maximum deviation distance is the distance between points B and C, and the deviation angle is the angle between the AB and AC lines. Point A is the coronal-most point of the fiber post outline on the same side as the maximum deviation location. Point B is the point of maximum deviation on the root canal wall, and point C is located on the fiber post outline at the same side and level as the maximum deviation

To measure the accuracy of each technique, 5 parameters were recorded comprising deviation volume, percent deviation volume, maximum deviation distance, deviation angle, and maximum deviation position. These parameters were defined as follows:

- Deviation volume is the amount of tooth structure removed after the fiber post removal procedure.
- Percent deviation volume is the percentage of volume of tooth structure removed divided by the total volume removed (the volume of tooth structure, fiber post and resin cement removed).
- Maximum deviation distance is the distance measured perpendicularly to the fiber post axis between the pre-operative root canal wall to the post-operative root canal wall in the area with the most tooth structure removed (Fig. 3).
- Deviation angle is defined as the angle between the AC and AB lines. Point A is the coronal most point of the fiber post outline on the same side as the maximum deviation location. Point B is the point of the maximum deviation on the root canal wall. Point C is located on the fiber post outline at the same side and level as the maximum deviation (Fig. 3).
- Maximum deviation position is the distance along the long axis of the canal from the coronal most level of the fiber post to the level where the maximum deviation distance occurs (Fig. 3).

These parameters were collaboratively measured by an engineer (S.K.) and a dentist (T.E.) who were blinded to the sample groups (Fig. 3). Efficiency was determined by the fiber post removal time recorded with a digital stopwatch.

### Statistical analysis

The deviation volume, percent deviation volume, maximum deviation distance, deviation angle, maximum deviation position and fiber post removal time were analyzed with the SPSS software package (IBM SPSS Statistics Version 29.0.1.0). The normality test was performed on the data using the Shapiro-Wilk test. The independent samples t-test was used when the data were normally distributed, and the Mann-Whitney U test was used when the data were skewed. A *p*-value < 0.05 was considered statistically significant.

## Results

Table 1 display the accuracy and efficiency parameters for the DNS-DOM and FH-DOM groups, as well as the parameters specific to tooth type (incisors, premolars, molars) within each group. The DNS-DOM group exhibited results that were similar to those of the FH-DOM group in deviation volume, percent deviation volume, maximum deviation distance, deviation angle, and maximum deviation position—factors all associated with accuracy. Considered by tooth type, we observed that the deviation volume and percent deviation volume of DNS-DOM, although not statistically different, were higher than those of FH-DOM in the incisors. In contrast, in the molars, these parameters were higher in the FH-DOM group compared with the DNS-DOM group. These results suggest that the free-hand technique is likely to cause less deviation when the operator has better visibility of the teeth. A trend of a higher deviation angle when using DNS-DOM compared with FH-DOM was observed across all tooth types. DNS-DOM and FH-DOM exhibited a higher deviation angle in molars compared with premolars and incisors, respectively. Moreover, the maximum deviation positions in molars were closest to the initial drilling points compared with those in incisors and premolars.

**Table 1:** The mean and standard deviation values of the deviation parameters determining accuracy

		DNS-DOM	FH-DOM
Deviation volume^X^ (mm^3^)	Overall	4.43 ± 2.99	4.36 ± 3.26
	Incisors	5.13 ± 3.59	3.16 ± 1.56
	Premolars	5.21 ± 2.89	5.74 ± 4.79
	Molars	2.97 ± 2.45	4.16 ± 2.75
Percent deviation volume^X^ (%)	Overall	30.59 ± 14.63	27.84 ± 14.68
	Incisors	34.95 ± 16.73	26.68 ± 14.70
	Premolars	32.30 ± 11.35	26.43 ± 17.11
	Molars	24.51 ± 16.34	30.42 ± 15.25
Maximum deviation distance^X^ (mm)	Overall	0.49 ± 0.19	0.49 ± 0.26
	Incisors	0.52 ± 0.21	0.47 ± 0.15
	Premolars	0.49 ± 0.07	0.49 ± 0.33
	Molars	0.47 ± 0.29	0.52 ± 0.32
Deviation angle (degree)	overall^Y^	10.45 ± 10.18	7.75 ± 6.00
	Incisors^X^	7.68 ± 3.47	6.65 ± 3.47
	Premolars^X^	8.67 ± 5.28	6.23 ± 4.53
	Molars^X^	15.01 ± 15.92	10.35 ± 8.86
Maximum deviation position^X^ (mm)	Overall	3.77 ± 1.91	4.35 ± 1.62
	Incisors	4.82 ± 1.38	4.57 ± 1.66
	Premolars	4.27 ± 2.28	5.08 ± 1.26
	Molars	2.23 ± 0.99	3.39 ± 1.71

^X^Independent samples t-test was used for the effects of fiber post removal methods on deviation volume, percent deviation volume, maximum deviation distance, deviation angle, and maximum deviation position. ^Y^Mann-Whitney U test was used for the effects of the fiber post removal methods on Overall deviation angle

Considering the efficiency of fiber post removal time, the DNS-DOM group demonstrated a longer mean procedure time compared with the FH-DOM group in all tooth types (mean difference (MD) = 480.93, *p* = 0.005; 95%, confidence interval (CI) = 158.97–802.90) with significant differences observed in the incisors (MD = 739.20, *p* = 0.005; 95%, CI = 175.71–1326.91) and molars (MD = 362.20, *p* = 0.005; 95%, CI = 19.75–704.65) (Table 2). Moreover, the trend of efficiency in fiber post removal was observed to be the highest in molars, moderate in premolars, and the lowest in incisors.

**Table 2:** The mean and standard deviation values of procedure time determining efficiency

	DNS-DOM	FH-DOM	Mean difference	95% CI
Overall	1334.73 ± 523.18 *	853.80 ± 296.86 *	480.93	158.97–802.90
Incisors	1762.80 ± 461.40 *	1023.60 ± 292.69 *	739.20	175.71–1326.91
Premolars	1261.40 ± 545.13	920.00 ± 237.21	341.40	-271.70–954.50
Molars	980.00 ± 234.30 *	617.80 ± 235.31 *	362.20	19.75–704.65

Asterisk indicates a significant difference between fiber post removal methods in each group using the independent samples t-test (*p* < 0.05). CI: confidence interval

## Discussion

This study demonstrated that, when performed by an experienced endodontist, the DNS-DOM technique offered similar accuracy, but lower efficiency, compared with FH-DOM in fiber post removal. Hence, we rejected only the null hypothesis that using the DNS-DOM and FH-DOM techniques for fiber post removal demonstrates similar efficiency. To our knowledge, one previous study has performed similar experiments and determined the deviation distance by measuring 2 parameters [6]. They evaluated global coronal deviation and global apical deviation that are the overall difference in position between the preplanned path and the actual drill path at the coronal and apical region, respectively. This method is reasonable because the drilling trajectory in the experimental group was done under the complete guidance of the navigation system without adjustment during the drilling process [6].

In our study, the drilling trajectory in the experimental group was influenced by using both DNS and a DOM. This led to the potential presence of multiple deviation trajectories in opposite directions, arising from mid-operation adjustments. When calculating the overall or global drilling trajectory, these opposing trajectories could negate each other, giving the impression of greater accuracy in the results. Hence, the deviation distance and angle in our study was assessed by comparing the pre-operative root canal wall with the post-operative root canal wall, as opposed to evaluating the global deviation trajectory. However, the previous study did include certain parameters comparable to ours, such as the operation time and the volume of tooth structure before and after the operation. These parameters can be subtracted to find the deviation volume.

Our study found that DNS-DOM had a longer procedure time than FH-DOM. However, a previous study found that FH-DOM had a longer procedure time than DNS alone [6]. There are several possible reasons for the different results between the 2 studies. In the previous study, the DNS group’s procedure followed the preplanned trajectory completely and did not require spending time looking through a DOM, resulting in a shorter procedure time [6]. In contrast, the operator in our study was performing under both DNS and DOM, requiring more time to process the extra information than completely following the preplanned trajectory as in the previous study [6]. The trend of efficiency in fiber post removal, measured by procedure time, was similar in both groups. Fiber post removal was fastest in molars and took progressively longer in premolars and incisors, indicating lower efficiency in these teeth (Table 1). The explanation might be that the increased size of the pulp chamber in posterior teeth allows the ultrasonic tip to enter at a wide range of angles, unlike the limited pulp chamber size in incisors and premolars. However, the larger pulp chamber also allowed the ultrasonic tip to deviate from the post axis earlier, resulting in closer maximum deviation positions to the initial drilling point and larger deviation angles in molars compared with incisors and premolars. However, a future study with a larger sample size for each tooth type needs to be conducted to confirm the significance of these observations.

The similar accuracy between the DNS-DOM and FH-DOM groups also contradicted the results from a previous study, which found that the DNS offered a significantly higher accuracy than the FH-DOM technique. However, it should be noted that, when comparing the measurements between our study and the previous study, the DNS-DOM and FH-DOM groups in our study displayed higher accuracy than the DNS and FH-DOM groups in the previous study, even though the fiber posts used in this study had a diameter of 0.9 mm at the apical tip and 1.12 mm at 7 mm from the tip, which is smaller than the parallel fiber posts with a diameter of 1.1 mm used in the previous study [6]. The most plausible explanation is the difference in the tip size of the drilling instruments and their cutting efficiency [6]. The previous study used #1 and #2 Munce burs with a diameter of 0.9 mm and 1.1 mm, respectively, while our study used the ET18D, a taper-shaped diamond-coated stainless steel ultrasonic tip with 76 μm diamond particles, a diameter of 0.7 mm at the tip, a length of 18 mm, and a 5% taper [6]. The smaller drilling tip might result in a smaller deviation. Furthermore, a Munce bur, a rotary instrument, has a much higher cutting efficiency than the ultrasonic tip, allowing faster removal of dentin. Consequently, a slight error in hand movement could result in a much higher loss of tooth structure in the previous study [6]. Moreover, it is possible that the small ultrasonic tip allowed the operator to better use the DOM and gain more control of the drilling instrument, increasing the accuracy in the DNS-DOM group and even more so in the FH-DOM group, rendering the difference between the groups non-significant. There were a case report which utilized a taper-shaped ultrasonic tip to successfully remove a fiber post under dynamic navigation [8].

The ultrasonic tip selection was based on the dentin cutting efficiency of ultrasonic tips reported in prior study that found that a diamond-coated ultrasonic tip has superior cutting efficiency than a stainless steel ultrasonic tip and an ultrasonic tip with micro projections [14]. Furthermore, our pilot study using ET18D, ET20D, ETBD, and CAP3 in fiber post removal demonstrated that ET18D had the highest efficiency. Our study used the recommended power setting for ET18D in endodontic treatment at 10 [15]. However, it should be noted that this power setting still provided low drilling power, occasionally forcing the operator to add extra force to help penetrate the fiber posts. A future study is planned to investigate the most suitable power setting for fiber post removal with ultrasonic tips.

It is also important to note that combining two different sets of instruments and techniques (DNS and DOM) can result in an unbalanced reliance on either instrument. Thus, to maximize the benefits from both the DOM and DNS, DOM was used for locating the fiber post, distinguishing the fiber post from the surrounding root canal wall, and confirming the ultrasonic tip’s position while the ultrasonic tip was not activated. Conversely, the DNS was relied on when the ultrasonic tip was activated, during which there would be debris and aerosol from the water coolant obscuring the field of operation under DOM. The accuracy of the DNS-DOM system also relies on the accuracy of feature point registration. The IRIS-100 manual recommended using apparent points on tooth structures as fiducial markers; however, our pilot study showed that the existing tooth structures, such as occlusal pits and cusp tips, could be ambiguous in the 3D reconstruction of the CBCT images during feature point registration planning. Therefore, our study decided to use an OGA with 4 fiducial markers, as recommended in previous IRIS-100 versions, during patient registration for the highest possible accuracy [16, 17]. The FOV of the CBCT used in this study was 80 × 80 mm, which was the smallest FOV capable of covering all OGAs while still providing the highest possible resolution. It is important to note that the FOV size does not significantly impact the accuracy of fiber post removal, as long as the voxel size is smaller than the fiber post [18]. This is supported by a previous study showing that CBCTs with both larger and smaller FOVs achieve comparable accuracy under these conditions [18].

Accuracy was further enhanced by placing the HT-JA-PAS on uninvolved teeth in the mandibular arch to reduce unintentional movement due to gravity. The HT portion was attached to the teeth using thermoplasticized resin, extending slightly under the height of contour of the teeth to help further stabilize the instrument. Moreover, because our study integrated the use of rubber dam sheets, HT-JA-PAS must be placed in an area with minimal tension from the rubber dam to reduce the chance of unintentional movement.

The strength of this study is that we used the DNS and DOM in a similar fashion to how they are used for fiber post removal in the clinical setting. Subsequently, the accuracy is a result of using as much information as possible from the DNS and DOM. Moreover, small ultrasonic tips were used to help achieve the highest possible accuracy in the fiber post removal procedure. This study provided a detailed approach to DNS registration and guidance in a simulated clinical setting, while also discussing solutions to minimize errors from the digital workflow, all of which could benefit future studies.

Our study incorporated several elements to simulate clinical conditions, including a mandibular cast, phantom head model, rubber dam, and consistent magnification. However, real-world variations such as operator experience, anatomical differences, and adjacent teeth could influence the generalizability of our findings. To control inter-operator variability, we used a single-operator design, though future studies should include multiple operators to assess performance across different skill levels. Additionally, we stratified our sample by tooth type to balance morphological differences and controlled for root curvature and diameter to ensure consistency. Further research is needed to evaluate these factors and the system’s efficacy under true clinical conditions.

Finally, since this study tried to simulate the clinical setting where the operator can adjust the drilling pathway, according to the available information, during the procedure, it is impossible to eliminate the influence of the endodontist’s experience. Therefore, additional investigations involving multiple operators with diverse clinical experience and varying years of experience are needed to investigate the impact of operator expertise on the accuracy of DNS in fiber post removal under DOM.

## Conclusion

This study demonstrates that, when performed by an experienced endodontist, DNS-DOM does not enhance the accuracy of fiber post removal compared with FH-DOM. Additionally, the navigation system requires extra operational time, potentially reducing procedural efficiency. To ensure methodological consistency and minimize inter-operator variability, a single operator was used in this study. However, future research involving multiple operators is necessary to further evaluate the generalizability of these findings to clinical practice.

## Data Availability

The data that support the findings of this study are available from the corresponding author upon reasonable request.

## Abbreviations

DOM: Dental operating microscope
DNS: Dynamic navigation system
IRIS: Implant Real-time Imaging System
FH: Freehand
CBCT: Cone-beam computed tomography
FOV: Field of view
HT: Holding tray
OGA: Occlusal guide appliance with four radiopaque fiducial markers
DICOM: Digital Imaging for Communication in Medicine
TU: Tracking unit
HRA: Handpiece reflective attachment
UHR: Ultrasonic handpiece registrator
JA: Jaw attachment
PAS: Positioner arm set
FPR: Feature point registration
MD: Mean difference
CI: Confidence interval
